# Personality Disorders in Functional and Idiopathic Dystonia

**DOI:** 10.3390/jcm15093544

**Published:** 2026-05-06

**Authors:** Violetta Aleksandrovna Tolmacheva, Vladimir Anatolyevich Parfenov, Dmitry Vladimirovich Romanov, Ekaterina Dmitrievna Spektor, Beatrisa Albertovna Volel, Ekaterina Vladimirovna Silina

**Affiliations:** Sklifosovskyi Institute of Clinical Medicine, Department of Nervous Diseases, I.M. Sechenov First Moscow State Medical University (Sechenov University), Trubetskaya St., 8, Moscow 119991, Russia; tolmacheva_v_a@staff.sechenov.ru (V.A.T.); parfenov_v_a@staff.sechenov.ru (V.A.P.); romanov_d_v@staff.sechenov.ru (D.V.R.); ekaterina.d.spektor@gmail.com (E.D.S.); volel_b_a@staff.sechenov.ru (B.A.V.)

**Keywords:** functional neurological disorder, functional dystonia, idiopathic dystonia, personality disorders, predictors of functional dystonia, SCID

## Abstract

**Background**: Distinguishing functional dystonia (FD) from idiopathic dystonia (ID) remains a major clinical challenge because both conditions are diagnosed primarily on clinical grounds and may be accompanied by non-motor psychiatric symptoms. Although personality abnormalities have been described in functional neurological disorders, their relevance in the differential diagnosis of dystonia remains insufficiently studied, and comparative data on FD and ID are lacking, particularly in the Russian population. **Patients and Methods**: A total of 178 patients with idiopathic dystonia (focal and segmental dystonia, ID) and 32 patients with functional dystonia (FD) were observed. A clinical interview by a psychiatrist was conducted; the SCID-II-PD questionnaire and the Five-Factor Personality Questionnaire (5-PFQ) were used to assess PD. **Results**: Patients with FD more often than patients with ID had such PD as dependent, paranoid, passive–aggressive, borderline, schizoid and schizotypal (*p* < 0.001), as well as obsessive–compulsive (*p* < 0.013) and avoidant (*p* < 0.049) according to SCID-II-PD. In FD, personalities of the eccentric cluster A predominate; patients with FD are characterized in personality terms by significantly greater introversion, detachment, naturalness (irresponsibility, impulsivity, carelessness), emotional restraint and practicality (conservatism, low sensitivity, rigidity) according to 5-PFQ. **Conclusions**: Patients with FD differ from patients with ID in both categorical and dimensional personality characteristics. The predominance of cluster A personality pathology and the identified pattern of personality-related variables may have potential value as adjunctive markers in the clinical differentiation of FD from ID. Further external validation is required before these findings can be incorporated into diagnostic algorithms.

## 1. Introduction

Functional movement disorders (FMDs) for many years were diseases that were classified as so-called “medical orphans.” For a number of reasons, such a diagnosis is difficult to establish without joint consultation of a neurologist and a psychiatrist [1,2,3,4,5]. The prevalence of FMD is quite high, from 80 to 140 per 100,000 people [6,7,8], and they are the second most common cause of a neurological outpatient visit after headache [9,10,11,12]. Among FMD phenotypes, functional dystonia (FD) remains one of the most diagnostically challenging presentations [1,2,13,14,15]. Taking into account the neuropsychiatric nature of idiopathic dystonia (ID) and diagnosis based on clinical criteria, it is extremely difficult to differentiate ID from functional dystonia (FD). Both ID and FD imply the absence of any anatomical, physiological, or biochemical abnormalities capable of explaining the occurrence of dystonia symptoms. In turn, the presence of comorbid non-motor psychiatric disorders (somatized, anxiety, depressive, delusional, etc.) causes even greater difficulties in differentiation with FD, taking into account the prevalence of these disorders in both discussed types of dystonia.

In the absence of reliable standards, the diagnosis of FD is often based on a clinical “gestalt” (from German “Gestalt”—form, image)—a spatial–visual form of generalized perception of the clinical picture as a whole, the essential properties of which cannot be understood only by summing individual symptoms [16].

The frequency of erroneous diagnosis of organic neurological disorders in earlier studies ranged from 25% [17] to 52% [18]. Although the diagnostic accuracy of FMD has since improved [19,20], dystonic manifestations remain a source of clinical errors [19,20,21].

The modern understanding of the genesis of dystonia requires a comprehensive assessment of mental status, which is not fully possible without taking into account personality characteristics, including personality disorders, because they may substantially influence symptom expression, coping strategies, and prognosis. It is known that personality patterns are a relatively stable characteristic of an individual, shaping behavior and responses to life events, including stress associated with somatic and neurological pathology. Studies evaluating personality disorders in functional disorders in neurology are few in number, study samples are small and use different psychometric instruments, and results are contradictory and not always comparable [22,23,24,25]. However, in terms of research hypotheses, the publications are united by the search for the role of personality characteristics in the development of functional disorders [26,27,28,29]. In addition, most published studies have focused on functional neurological disorders in general rather than on direct comparison between functional dystonia and idiopathic dystonia. Thus, the specific contribution of personality disorders and dimensional personality traits to the differentiation of FD from ID remains insufficiently understood. Data on personality disorders in functional and idiopathic dystonia in the Russian population are absent.

Therefore, the present study aimed to compare the prevalence and structure of personality disorders and dimensional personality traits in patients with functional dystonia versus idiopathic dystonia and to evaluate whether personality-related variables may contribute to the practical diagnostic differentiation of FD and ID.

## 2. Materials and Methods

### 2.1. Patients

A single-center cross-sectional study was conducted, including patients with focal and segmental dystonia. The study included patients with idiopathic focal and segmental dystonia diagnosed according to the Albanese criteria [30]. The study also included patients with FDR (*n* = 32), simulating various forms of dystonia and specifically mimicking focal or segmental dystonia (according to DSM-5-TR [31] criteria and clinically established reliability criteria according to Fahn and Williams, 1988; Lang A.E. 2011 [32]), who sought outpatient care at the Clinic of Nervous Diseases named after A.Ya. Kozhevnikov of Sechenov University (Moscow, Russia).

### 2.2. Methods

Each patient underwent examination including clinical neurological examination and a clinical psychiatric interview to assess complaints, medical history data, life history, current psychopathological symptoms, and psychiatric comorbidity; however, because of the heterogeneity of comorbid conditions and the limited size of the FD group, these variables were not entered as separate covariates into the final models.

To assess personality disorders (PDs), the SCID-II-PD questionnaire was used [33]. It is a structured clinical interview designed to diagnose DSM-IV Axis II personality disorders, including avoidant, dependent, obsessive–compulsive, paranoid, schizotypal, schizoid, histrionic, narcissistic, borderline, antisocial, passive–aggressive/negativistic, and self-defeating personality disorders. The instrument consists of 119 questions concerning the subject’s opinion about their personal traits, divided into 12 sections intended for self-completion, followed by a diagnostic interview with a psychiatrist. A Russian-language clinical version used in routine psychiatric practice was applied by the co-author psychiatrists D.V.R. and B.A.V. Final personality disorder diagnoses were established by consensus between the two psychiatrists.

The Five-Factor Personality Questionnaire (5-PFQ) was also used, which consists of 75 statements opposite in meaning, each identifying important personality traits and characteristics of human behavior in certain life situations. The questionnaire characterizes generalized trait factors and their primary components based on the approach to the five-factor personality structure. The method was adapted by the Japanese personality researcher Hidzhiro Tsuji based on the developments of P. Costa and R. McCrae (Neo PI-R questionnaire). He proposed bipolarity for each factor, extraversion–introversion, attachment–detachment, self-control–impulsivity, emotional instability–emotional restraint, and expressiveness–practicality, which makes it possible to characterize personality, since each pole of a trait reflects its peculiarity. The domestic adaptation was performed by A.B. Khromov at Kurgan State University in 2000 [34].

Missing data were minimal (<5% across questionnaires). If ≤10% of items within a subscale were missing, values were imputed using the participant’s mean score for the remaining items of that subscale. Questionnaires with >10% missing responses per subscale were excluded from analysis.

For SCID-II-PQ, items were scored dichotomously (0/1), and subscale scores represented the sum of endorsed criteria. Higher scores indicated greater expression of the respective personality disorder traits. A threshold of >6 points was used to define a pathological result.

For the 5-PFQ, secondary factor scores were calculated according to the validated scoring key. The instrument uses bipolar dimensions; higher values correspond to the first pole of each factor (e.g., extraversion, attachment, control, emotionality, playfulness), whereas lower values correspond to the opposite pole (introversion, detachment, naturalness/impulsivity, emotional restraint, practicality).

### 2.3. Statistical Analysis

Statistical analyses were performed using the R programming language (version 4.4.1) within the RStudio environment (v. 2025.09.0+387, Posit Software, PBC, Boston, MA, USA). Continuous variables were compared using the Mann–Whitney U test, whereas categorical variables were analyzed using the chi-square test, given the unequal group sizes and the distributional characteristics of the data.

The association between functional dystonia and specific personality disorders was evaluated using logistic regression analysis, with the presence of functional dystonia as the dependent variable, and predictors were the score for each SCID subscale. Class imbalance (the studied sample included 178 patients with ID and 32 with functional dystonia) was corrected by weighting: weights were assigned in such a way as to balance representation of the smaller class and increase its influence on the model, while the observed age difference between groups was taken into account during interpretation of the findings as a potential confounding factor. The model was constructed using the glm-function in the R basic syntax.

At the first step, regression models with one predictor were constructed for each of the 12 SCID subscales (avoidant, dependent, obsessive–compulsive, passive–aggressive, depressive, paranoid, schizotypal, schizoid, histrionic, narcissistic, borderline, antisocial disorder). Further, only those predictors whose influence was significant in single-predictor models (scores of avoidant, dependent, obsessive–compulsive, passive–aggressive, paranoid, schizotypal, schizoid, narcissistic, borderline, and antisocial disorder) were included in the full multiple regression model; thus, the initial full model included 10 predictors.

This model was subjected to analysis for predictor multicollinearity using the vif() function from the car package, according to the results of which no sources of pronounced multicollinearity in the model were detected (vif < 2 for each predictor). Then statistically non-significant predictors were stepwise removed from the model until all predictors included in the multiple regression model had a significance level *p* for the coefficient less than 0.05. For quantitative assessment of predictor influence, their standardization was performed.

ROC analysis of the classifier derived from the final model was performed using the pROC package in R. The roc() function was used to construct the ROC object, and the ci() function was applied to calculate the confidence interval for the area under the curve (AUC) using DeLong’s method. All statistical tests were two-sided, and *p* < 0.05 was considered statistically significant.

To visualize the diagnostic algorithm allowing differentiation between functional and idiopathic dystonia based on SCID data, a decision tree was constructed using the predictors selected at the previous stage. It is important to emphasize that this tree was used solely as a tool for visualizing the diagnostic decision logic rather than as an independent machine learning algorithm for prediction. The tree was implemented in Python 3.10 (Python Software Foundation, Wilmington, DE, USA) using the numpy (v. 2.2.6, Python Software Foundation, Wilmington, DE, USA), pandas (v. 2.2.2, Python Software Foundation, Wilmington, DE, USA), and scikit-learn (v.1.7.0, Python Software Foundation, Wilmington, DE, USA) libraries. Since patients with functional dystonia represented only 15.2% of the sample (32 vs. 178 patients with idiopathic dystonia), bootstrap replication was applied to address class imbalance. From the original FD group (*n* = 32), four bootstrap samples of the same size were generated using random sampling with replacement (seed = 17) and subsequently combined with the original dataset. This approach increased the representation of the FD class in the training data while preserving the original distribution of features within the group. The dataset was then divided into training and test sets using stratified random sampling (test_size = 30, random_state = 17). The training set contained 262 observations after bootstrap augmentation, while the test set included 30 observations with the original class proportions preserved. The decision tree model was trained using the entropy criterion. To prevent overfitting and ensure clinical interpretability, the following constraints were applied: maximum tree depth of five levels (based on the number of predictors), a minimum of 30 samples required to split a node, and a minimum of 10 samples in a leaf node. Model robustness was evaluated using five-fold cross-validation on the training dataset.

## 3. Results

The most clinically relevant findings were the higher frequency of cluster A-related personality pathology in FD, the identification of schizoid, schizotypal, paranoid, dependent, and borderline traits as the main predictors of FD, and the decision-tree model illustrating the practical differentiation between FD and ID.

### 3.1. Patient Characteristics

The study included 210 patients, of whom 178 patients had idiopathic dystonia (ID, 84.7%) and 32 patients had functional dystonia (FD, 15.2%). In the ID group, dystonia phenotypes included blepharospasm (*n* = 50), cervical dystonia (*n* = 85), cranial dystonia (*n* = 35), and writer’s cramp (*n* = 8). In the FD group, the corresponding functional presentations were blepharospasm (*n* = 7), cervical dystonia (*n* = 12), cranial dystonia (*n* = 9), and writer’s cramp (*n* = 4). The proportion of men (56 persons (31.5%) in the ID group, 10 persons (31.2%) in the FD group) did not differ significantly between the groups (*p* = 1), whereas the subjects from the FD group were on average younger (41.2 (13.3) years versus 56.5 (12) years, *p* < 0.001), which was taken into account in the interpretation of the findings as a potential confounding factor.

#### 3.1.1. Pathocharacterological Profiles of ID and FD According to the Frequency and Severity of Categorical Personality Disorders According to SCID-II

**Figure 1:**
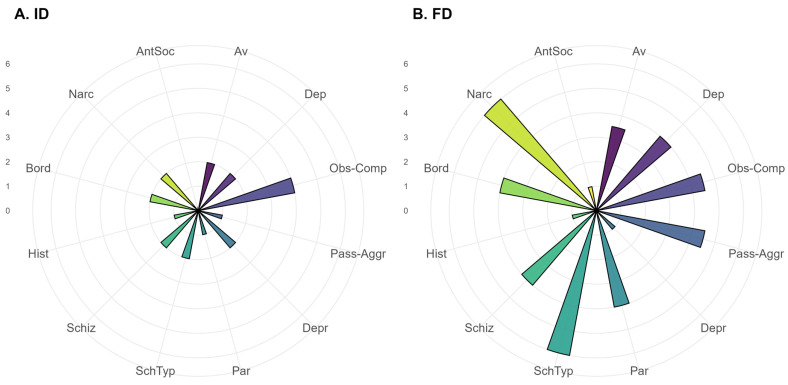
Profiles of patients of the (**A**) ID and (**B**) FD groups according to the SCID-II questionnaire, depending on the sum of scores obtained on individual personality disorder subscales. Notes: Av—avoidant, Dep—dependent, Obs-Comp—obsessive–compulsive, Pass-Aggr—passive–aggressive, Depr—depressive, Par—paranoid, SchTyp—schizotypal, Schiz—schizoid, Hist—histrionic, Bord—borderline, Narc—narcissistic, AntSoc—antisocial.

**Figure 2:**
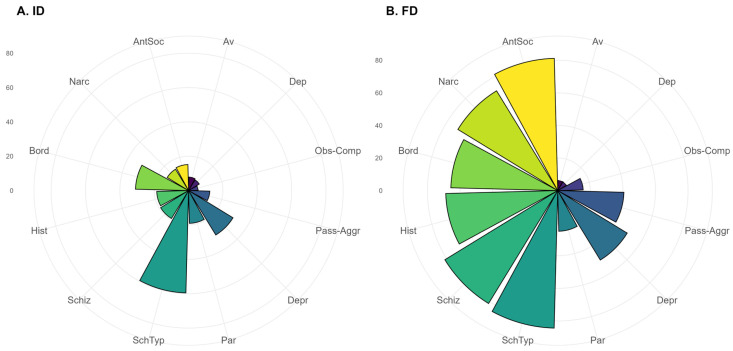
Profiles of patients of the (**A**) ID and (**B**) FD groups according to the SCID-II scale by the proportion of patients with pathological results for various personality disorders. Notes: Av—avoidant, Dep—dependent, Obs-Comp—obsessive–compulsive, Pass-Aggr—passive–aggressive, Depr—depressive, Par—paranoid, SchTyp—schizotypal, Schiz—schizoid, Hist—histrionic, Bord—borderline, Narc—narcissistic, AntSoc—antisocial.

The absolute number and proportions of patients having a pathological result for each of the SCID-II subscales > 6 points, as well as comparative analysis of proportions, are presented in Table 1. Patients with FD statistically significantly more often have the following disorders: dependent, avoidant, obsessive–compulsive, paranoid, passive–aggressive, borderline, schizoid, and schizotypal. Figure 1 and Figure 2 present the profiles of patients of the two groups according to the SCID-II scale from the standpoint of the sum of scores obtained on the subscales (Figure 1, the bar height reflects the median value) and according to the proportion of patients with pathological results on the subscales (Figure 2; the bar height reflects the percentage of pathological results in the group).

The final regression model obtained as a result of the study contained, as predictors of FD, scores on the SCID subscales for dependent, paranoid, schizotypal, schizoid, and borderline personality disorders, which represented the most clinically informative variables in the model. The results of the analysis, including regression coefficients, standard errors, and odds ratios, as well as statistical significance of the predictors, are presented in Table 2. To assess the classification quality using the obtained model, ROC analysis was performed with the determination of the area under the curve.

**Figure 3:**
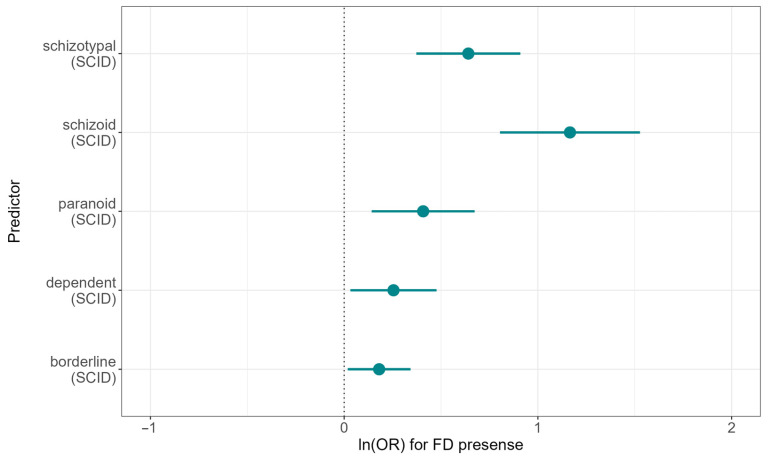
Predictors of FD according to the SCID scale based on logistic regression analysis. Along the *X*-axis—the logarithm of the odds ratio (point—estimate of the regression coefficient for the corresponding predictor; segment limits—confidence interval limits). Along the *Y*-axis—predictors in the logistic regression model (the vertical dashed line indicates the logarithm of the odds ratio equal to 0, which corresponds to an odds ratio equal to 1 or absence of significance).

The results of the analysis with standardized predictors show that the contribution of scores on the SCID-II subscales to the probability of FD is ranked as follows: schizoid (OR 1.17, 95% CI 0.8–1.53) > schizotypal (OR 0.64, 95% CI 0.37–0.91) > paranoid (OR 0.41, 95% CI 0.14–0.67) > borderline (OR 0.18, 95% CI 0.02–0.34) > dependent disorder (OR 0.25, 95% CI 0.03–0.48). The logarithm of the odds ratio for each of the factors is presented in Figure 3.

**Figure 4:**
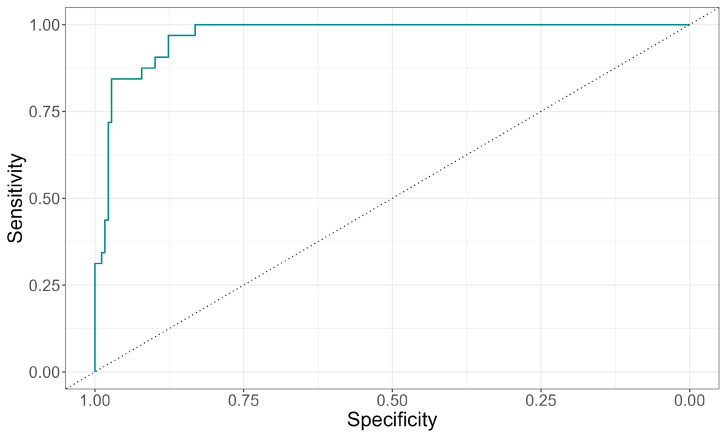
ROC curve for the classifier based on the constructed logistic regression model.

When assessing the classification quality based on the obtained model using ROC analysis, the area under the curve was 0.97 (95% CI 0.95–0.99), which corresponds to good classifier quality (Figure 4).

#### 3.1.2. Construction of a Decision Tree Taking into Account Significant Predictors According to SCID-II

**Figure 5:**
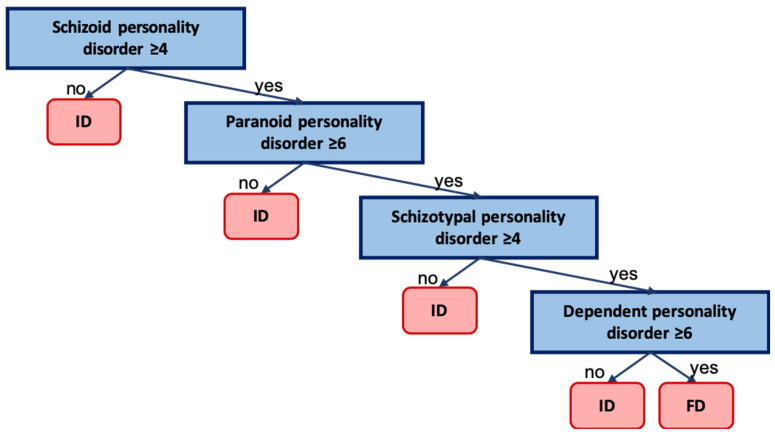
Decision tree. The tree nodes correspond to SCID subscale values above or below the diagnostic threshold for each personality disorder, which determines the branching direction. ID—idiopathic dystonia; FD—functional dystonia.

Following the identification of significant predictors through logistic regression analysis, a decision tree was constructed as a tool for graphical representation of the decision-making logic for differentiating between functional and idiopathic dystonia with emphasis on its potential clinical applicability. The obtained classification algorithm (Figure 5) had an accuracy of 83.7%, a sensitivity of 90.6%, and a specificity of 77.5%.

The decision tree included the SCID-II predictors retained in the final model and provided a visual representation of the classification process. Each node of the tree contains a condition indicating whether the score on a particular subscale exceeds the threshold value for one of the listed personality disorders. The corresponding terminal nodes indicate classification as FD or ID.

For comparative analysis of the results of the five-factor questionnaire, taking into account the asymmetric distribution of variables, the nonparametric Mann–Whitney test was used. Descriptive statistics and results of comparison are presented in Table 3.

**Figure 6:**
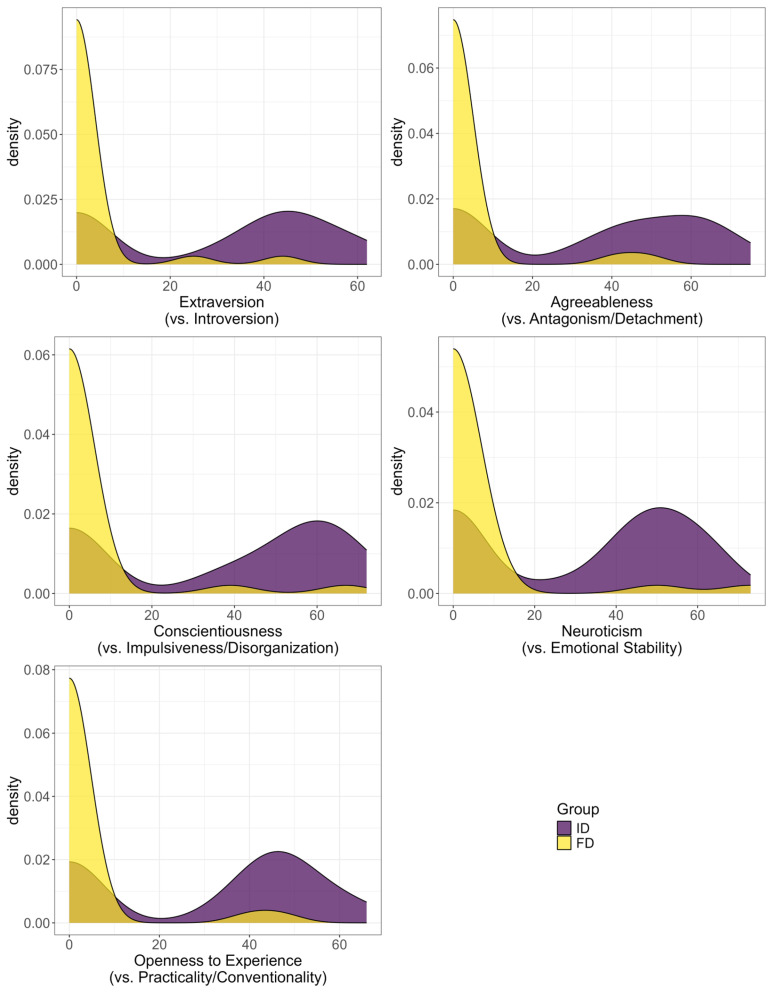
Distribution of values of indicators of secondary factors of the five-factor personality questionnaire in the study groups.

Differences were identified in patients with FD for each of the factors. Thus, patients with FD showed significantly greater introversion (withdrawal, avoidance of new experiences), detachment (aloofness, indifference, suspiciousness), naturalness (irresponsibility, impulsivity, carelessness), emotional restraint (self-sufficiency), and practicality (conservatism, low sensitivity, rigidity). Patients with ID showed lower practicality (sensitivity, dreaminess), greater emotionality (anxiety, depressiveness, self-criticism), and extraversion (activity, sociability). The obtained dimensional profile of psychological characteristics was consistent with the between-group differences observed on SCID-II. These differences are illustrated in Figure 6.

## 4. Discussion

In the study conducted by us, it was shown that patients with FD, in categorical assessment of the personality profile using SCID-II, statistically significantly more often, compared with ID, have the following personality disorders: dependent, avoidant, obsessive–compulsive, paranoid, passive–aggressive, borderline, schizoid, and schizotypal. At the same time, the highest total scores on individual SCID-II subscales are determined for schizotypal and narcissistic personality disorders. In the available literature, there are few publications devoted to the direct comparative assessment of types of personality disorders and personality traits in FD and ID. Existing studies on personality characteristics in dystonia mainly reveal only differences in personality profiles of dystonia as a whole, without differentiation into subtypes, compared with healthy controls without neurological disorders. For example, in the publication by Davidescu et al., assessment using the DECAS personality questionnaire revealed that patients with dystonia were significantly more often prone to fantasizing (*p* = 0.007), experimentation (*p* = 0.022), and sophistry (*p* = 0.040), but less often prone to acceptance (*p* = 0.022) and pragmatism (*p* = 0.022) compared with neurologically healthy participants of the control group [35].

In addition, among publications on the discussed problem, there are studies devoted to identifying personality characteristics of certain focal forms of dystonia compared with others. Thus, in the study by Steinlechner et al., when comparing “musician’s dystonia” (*n* = 101) with other focal dystonias (*n* = 85) using the NEO-FFI personality questionnaire, it was established that “musician’s dystonia” is characterized by a specific personality profile with increased levels of neuroticism and openness compared with other isolated focal dystonias [36].

In the study by Luca A et al., analysis of personality and psychopathological characteristics among patients with functional movement disorders (predominantly tremor and muscle weakness) and other neurological diseases was performed; however, no statistically significant differences in personality assessment were identified. At the same time, the authors note that patients with functional movement disorders are characterized by “conformist behavior,” a maladaptive avoidant behavioral style, and difficulties in coping with emotional stress, which may contribute to the development of the disease [24].

In the study by Tomić A et al., a comparative analysis was presented between a group of patients with functional dystonia (39 cases) and a case–control matched group of patients (30 cases) with idiopathic (“primary”) dystonia with respect to such characteristics as the frequency and spectrum of psychiatric disorders, psychological stressors, dissociative disorders, and personality traits [37]. The authors established that FD is characterized by lower levels of extraversion and openness to experience; i.e., patients with FD were more introverted and more closed toward new experiences. In general, characterizing the group of patients with FD, Tomić et al. noted such traits typical for them as emotional detachment, restraint, and unsociability [38], which in fact corresponds to the personality characteristics of cluster A (detached or “estranged” personalities) identified in our study. The authors indicate that cognitive processes in these patients are formally not impaired; thinking is rational, logical, and pragmatic but devoid of imagination and fantasy, which relates to the concept of “operant thinking” [39]. These results indicate that the emotional life of patients with FD is less differentiated and corresponds to the concept of alexithymia (impairment of emotional processing at the cognitive level)—a typical deficit in conversion disorders [40].

The role of personality disorders as a risk factor in the development and maintenance of functional movement disorders has been noted in several studies [19,41]; however, to date, no study has demonstrated a higher prevalence of schizoid, paranoid, and schizotypal personality disorders in patients with FD. Feinstein et al. found that the prevalence of personality disorders was 42% in a sample of patients suffering from functional movement disorders; these were mainly antisocial, borderline, and dependent personality disorders. Similar results were obtained by Howarka J. [41] and Reuber M. et al. [42] in patients with psychogenic nonepileptic seizures: both studies identified high rates of borderline personality disorder. Demartini B. et al. demonstrated a significant proportion of obsessive–compulsive personality disorder in patients with functional movement disorders compared with patients with organic movement disorders and healthy controls [40]. On the other hand, Kranick S. et al. [43], assessing personality traits in a population of patients with functional movement disorders using the Revised Neuroticism–Extraversion–Openness Personality Inventory, did not identify any significant differences in their patient group compared with healthy controls. Such inconsistency among study findings may be related to the fact that different forms of functional movement disorders (pareses, various variants of hyperkinesias) may be associated with different personality types (assessed using categorical instruments).

The personality disorders identified by us as the most significant predictors of FD—schizoid, paranoid, and schizotypal personality disorders—belong to a single personality disorder cluster according to DSM, namely the eccentric (oddy) cluster A. This cluster is characterized by difficulties in interpersonal communication, emotional coldness, detachment, and inability to establish close interpersonal relationships (formality of contacts), as well as excessive suspiciousness toward the surrounding environment without sufficient grounds. In turn, schizotypal and dependent personality disorders are classified by a number of researchers in personality psychopathology as among the most severe personality disorders (along with borderline personality disorder) [44,45,46]. Thus, according to the obtained data, FD is characterized by a personality profile in which accumulation of severe personality disorders is observed, as well as pathocharacterological abnormalities within eccentric cluster A.

We performed an analysis of the results of the five-factor questionnaire. The identified differences showed that patients with FD are characterized, in personality terms, by substantially greater introversion (withdrawal, avoidance of new impressions), detachment (aloofness, indifference, suspiciousness), naturalness (irresponsibility, impulsivity, carelessness), emotional restraint (self-sufficiency), and practicality (conservatism, low sensitivity, rigidity), which, as indicated above, is consistent with the work of Tomić A. et al. Compared with patients with FD, patients with ID showed lower practicality (sensitivity, dreaminess), greater emotionality (anxiety, depressiveness, self-criticism), and greater extraversion (activity, sociability). The obtained dimensional profile of psychological characteristics corresponds to SCID-II data, according to which eccentric cluster A personalities predominate in FD.

According to the data obtained by us, the distribution of results for each personality factor is characterized by bimodality; that is, it has two peaks. This reflects the presence of two poles of each factor, which logically follows from the nature of the phenomenon assessed by it: the studied personality characteristics represent a binary opposition, and each examined individual can be attributed to one of the poles (extraversion or introversion, attachment or detachment, and so on), taking into account some variability in the degree of expression of each personality characteristic. It is evident that patients with FD as a whole are characterized by an imbalance of peaks with a sharp predominance, for each factor, of the peak in the region of low scale values, whereas in the other groups, the probability density of both peaks is more balanced.

Personality is essentially a stable internal factor ensuring the consistency of individual behavior. In addition, personality traits play a fundamental role in stress resistance, coping effectiveness, and interpersonal interaction [47,48,49]. Personality disorders represent a significant premorbid parameter and an important factor of predisposition to many mental disorders.

Characterological anomalies (psychopathies, personality disorders), as a group of relatively stable disorders, beginning with E. Kraepelin and subsequently E. Kretschmer, A.V. Snezhnevsky, A.B. Smulevich, and other psychiatrists who adhered in their studies to the medical model of psychopathies [46,50,51], were considered as the rudiments of a future disease, as a premorbid condition indicating a potential possibility of manifestation of a pathological psychopathological process, that is, from modern positions, as a risk or vulnerability factor, including with respect to the development of functional and other psychosomatic disorders. Consequently, individualization of personality characteristics may be useful for an individualized approach during diagnosis and for the selection of methods of individualized combined therapy of dystonia.

The practical significance of the conducted study may be associated with the following. We proposed a decision tree as an exploratory tool for formalizing the process of diagnostic reasoning and determining the possible functional nature of dystonia on the basis of objective psychometric data. Each node of the tree contains a condition indicating whether the score on a specific subscale exceeds the threshold value for one of the listed personality disorders. Depending on the answer (“Yes” or “No”), movement to the next node is performed, where it is again necessary to answer a question related to another subscale or characteristic, or this node will be terminal; that is, it will contain a formal conclusion about the possible nature of dystonia (functional or idiopathic). If a patient has a high score on each of the subscales appearing in the tree nodes (schizotypal, schizoid, paranoid, and dependent personality disorders), the probability that dystonia has a functional origin is high. In the absence of such elevation (in accordance with the specific threshold indicated in the node condition) in at least one of the subscales, the probability of idiopathic dystonia is higher.

Our study is based on the comparison of patients with FD and ID, whereas in other studies, personality disorders in various functional neurological disorders were investigated without comparison with ID [20,31].

A limitation of our study was the relatively small number of patients with FD compared with ID, which reflected the study design, since the FD group was intentionally restricted to patients with functional presentations mimicking focal or segmental dystonia, and which did not allow the formation of a test sample and the assessment of the quality of the model performance. In addition, the prevalence of FD among all dystonias is probably lower than in the sample studied by us; therefore, the proposed algorithm may produce estimates biased toward FD, and external validation in real clinical practice is required to identify such a problem, while the observed group proportions should be interpreted as reflecting the structure of the selected clinical sample rather than population prevalence. Another limitation is the absence of prospective follow-up of patients with FD with regard to the dynamics of the clinical presentation of the motor deficit. In addition, the younger age of the FD group and the possible confounding effect of psychiatric comorbidity cannot be fully excluded and should be taken into account when interpreting the observed between-group differences. An additional limitation is that, although final personality disorder diagnoses were established by consensus between two psychiatrists, formal inter-rater reliability was not quantified.

## 5. Conclusions

These conclusions should be interpreted with caution given the cross-sectional design, the relatively small size of the FD group, the age difference between groups, and the possible confounding effect of psychiatric comorbidity

In the existing diagnostic criteria for functional neurological disorders in neurological practice, the necessity of assessing psychological and personality factors in patients is not specified. Our data indicate the presence of certain personality patterns in patients with FDs compared with patients with IDs. In FDs, more often than in IDs, such PDs as dependent, paranoid, passive–aggressive, borderline, schizoid and schizotypal, obsessive–compulsive and avoidant are observed; personalities of the eccentric cluster A predominate, which may represent clinically relevant associated features and may have potential value as adjunctive markers in the differentiation of FD from ID, although these findings should not be interpreted as established diagnostic criteria and require external validation.

In the era of technological progress in medicine, enormous advances have been achieved in diagnostic methods in such fields as genetics, electrophysiology, and imaging. This provides substantial assistance but should not lead to underestimation of the importance of clinical examination. In particular, in the field of functional neurological disorders, semiology and clinical examination retain primary importance and represent a reliable way of establishing the diagnosis. Future multimodal diagnostic algorithms combining semiology, electrophysiological imaging, and biomarkers may increase diagnostic reliability, which will further improve clinical care and research opportunities in this field. At present, testing of these positive signs at the bedside not only increases diagnostic accuracy but also allows the establishment of contact with the patient, which is an important first step toward treatment. Clarification of the contribution of various neurocognitive mechanisms, in particular through personality disorders in FD, may in the future help to identify FD biomarkers or clinically useful supportive indicators guiding treatment selection and determining prognosis and help patients better understand their condition and reduce the stigmatization associated with the diagnosis of a functional disorder.

## Figures and Tables

**Table 1:** Absolute number and proportions of patients having a pathological result for each of the SCID-II subscales > 6 points, as well as comparative analysis.

SCID Subscale	Idiopathic Dystonia, N = 178	Functional Dystonia, N = 32	χ^2^	*p*-Value
Antisocial, *n* (%)	14 (7.9%)	2 (6.2%)	0	1
Histrionic, *n* (%)	13 (7.3%)	2 (6.2%)	0	1
Depressive, *n* (%)	10 (5.6%)	5 (15.6%)	2.7	0.1
Dependent, *n* (%)	22 (12.4%)	13 (40.6%)	13.6	<0.001
Avoidant, *n* (%)	54 (30.3%)	16 (50%)	3.9	0.049
Narcissistic, *n* (%)	34 (19.1%)	8 (25%)	0.28	0.6
Obsessive–compulsive, *n* (%)	106 (59.6%)	27 (84.4%)	6.17	0.013
Paranoid, *n* (%)	34 (19.1%)	26 (81.2%)	48.3	<0.001
Passive–aggressive, *n* (%)	33 (18.5%)	22 (68.8%)	32.8	<0.001
Borderline, *n* (%)	55 (30.9%)	21 (65.6%)	12.7	<0.001
Schizoid, *n* (%)	26 (14.6%)	23 (71.9%)	46.5	0.001
Schizotypal, *n* (%)	27 (15.2%)	26 (81.2%)	59.3	<0.001

**Table 2:** Characteristics of the regression model evaluating the significance of pathocharacterological predictors according to SCID in the development of FD.

Predictor (SCID)	Coefficient β	Standard Error β	Odds Ratio (OR)	*p*-Value
Dependent disorder	0.25	0.11	1.29	0.025
Paranoid disorder	0.41	0.13	1.5	0.002
Schizotypal disorder	0.64	0.14	1.9	<0.001
Schizoid disorder	1.17	0.18	3.2	<0.001
Borderline disorder	0.18	0.08	1.2	0.029

**Table 3:** Comparison of indicators of secondary factors of the five-factor personality questionnaire in the study groups. M (sd)—mean value and standard deviation; ID—idiopathic dystonia; FD—functional dystonia.

Factor	Idiopathic Dystonia, *n* = 178	Functional Dystonia, *n* = 32	*p*-Value
Extraversion—introversion (higher values correspond to extraversion), M (sd)	28.7 (23.6)	2.1 (8.8)	<0.001
Attachment—detachment (higher values correspond to attachment), M (sd)	33.2 (27.6)	2.8 (11.1)	<0.001
Control—naturalness (higher values correspond to control), M (sd)	35.2 (28.6)	3.3 (13.5)	<0.001
Emotionality—emotional restraint (higher values correspond to increased emotionality), M (sd)	31 (25.6)	3.8 (15.4)	<0.001
Playfulness—practicality (higher values correspond to playfulness), M (sd)	30.1 (24.4)	2.7 (10.7)	<0.001

## Data Availability

Data will be made available upon reasonable request by the corresponding author. Data are not publicly available due to privacy or ethical restrictions.
